# BK Virus Infection and BK-Virus-Associated Nephropathy in Renal Transplant Recipients

**DOI:** 10.3390/genes13071290

**Published:** 2022-07-21

**Authors:** Margherita Borriello, Diego Ingrosso, Alessandra Fortunata Perna, Angela Lombardi, Paolo Maggi, Lucia Altucci, Michele Caraglia

**Affiliations:** 1 Department of Precision Medicine, University of Campania “Luigi Vanvitelli”, Via L. De Crecchio 7, 80138 Naples, Italy; margherita.borriello@unicampania.it (M.B.); diego.ingrosso@unicampania.it (D.I.); angela.lombardi@unicampania.it (A.L.); lucia.altucci@unicampania.it (L.A.); 2 Department of Translational Medical Science, University of Campania “Luigi Vanvitelli”, Via Pansini, Bldg., 80131 Naples, Italy; alessandra.perna@unicampania.it; 3 Department of Mental and Physical Health and Preventive Medicine, University of Campania “Luigi Vanvitelli”, Via L. De Crecchio 7, 80138 Naples, Italy; paolo.maggi@unicampania.it; 4 Laboratory of Precision and Molecular Oncology, COVID19 Laboratory, Biogem Scarl IRGS, Via Camporeale, 83031 Ariano Irpino, Italy

**Keywords:** BK virus infection, renal transplanted recipients, BKV nephropathy, miRNA, biomarkers, urine biomarkers, early diagnosis

## Abstract

Poliomavirus BK virus (BKV) is highly infective, causing asymptomatic infections during childhood. After the initial infection, a stable state of latent infection is recognized in kidney tubular cells and the uroepithelium with negligible clinical consequences. BKV is an important risk factor for BKV-associated diseases, and, in particular, for BKV-associated nephropathy (BKVN) in renal transplanted recipients (RTRs). BKVN affects up to 10% of renal transplanted recipients, and results in graft loss in up to 50% of those affected. Unfortunately, treatments for BK virus infection are restricted, and there is no efficient prophylaxis. In addition, consequent immunosuppressive therapy reduction contributes to immune rejection. Increasing surveillance and early diagnosis based upon easy and rapid analyses are resulting in more beneficial outcomes. In this report, the current status and perspectives in the diagnosis and treatment of BKV in RTRs are reviewed.

## 1. BK Virus

**Figure 1:**
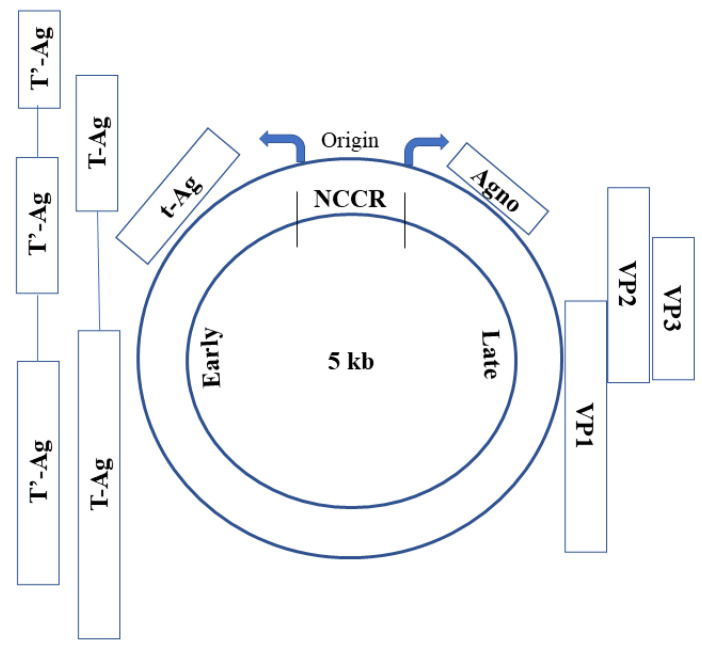
Graphical Sketch of BKV Genome. Early Genes Region: T-Ag (Large T antigen), T’-Ag (alternatively-spliced T-Ag), t-Ag (small T antigen); NCCR: Non-Coding Control Region; Late Genes Region: Agno (Agnoprotein), VP1–3 (viral capsid proteins).

BK virus (BKV) is an icosahedral virus with a double-stranded DNA genome. It is a member of the polyomavirus (PV) family, together with John Cunningham virus (JC) and Simian virus 40 (SV40). The BKV genome (≈5 kb) is divided into: (i) the early region (which codes for small (t) and large T-antigen); (ii) the late region (which codes for capsid proteins Vp1, Vp2, Vp3, agnoprotein and microRNAs) and (iii) the non-coding control region (NCCR) (Figure 1) [1].

BKV strains are classified into six genotypes, according to polymorphisms in VP1 and NCCR [1], with genotype I frequency at around 80% and genotype IV at 15% [2]. BKV infects most of the world population in their youth, often with silent infections (i.e., without symptoms) [3]. After the first infection, a kind of inactive infection is established in kidney and uroepithelium [4,5,6]. Generally, BKV transmission occurs through respiratory secretions [7]. In particular, after infection, BKV is able to shape intranuclear inclusion bodies of 40–45 nm in size in the renal tubular cells [8]. Moreover, the periodical and transient presence of BKV in the urine of immunocompetent adults has been demonstrated (between 5% and 27%), as a consequence of its shedding [9].

Viral agnoprotein seems to have a key role in the infective cycle of BKV. Agnoprotein is expressed in some polyomaviruses [10]. BK, JC and SV40 agnoproteins have a sequence identity of up to 83%, suggesting a potential shared function. Agnoprotein is cytoplasmic or perinuclear during the tardive stages of the PV cycle [11]. It has recently been demonstrated that agnoprotein co-localize with lipid droplets in BK infected primary renal tubular epithelial cells [12]. Nevertheless, the importance of this finding is still unclear. It was pointed out that agnoprotein is mostly found at the later stages of the polyomavirus life cycle. Therefore, it has been proposed that agnoprotein assumes a key role in virion assembly, morphogenesis and release. Agnoprotein is not crucial for virion infectivity or morphogenesis, even if its absence renders viruses unable to release from host cells and propagate. Instead, agnoprotein expression is related to the exit of BK virions from the nucleus, increasing the speed of the viral cycle. The α-soluble, *n*-ethylmaleimide-sensitive fusion (NSF) attachment protein α-SNAP is the binding moiety of agnoprotein, and is essential for BK virion release [13]. Another important function of agnoprotein is its involvement in the induction of immunological escape, contributing to viral persistence. In fact, it was recently reported that BK destroys mitochondrial interaction and reduces mitochondrial membrane potential upon the expression of the 66 aa-long agnoprotein during late replication. This effect is paralleled by the impairment of IRF3 transportation in the nucleus and of interferon-beta levels, and by the induction of p62/SQSTM1 mitophagy. These in vitro effects were confirmed by the observation of mitochondrial degradation and by the increase of the autophagic marker p62/SQSTM1 in allograft biopsies of kidney transplanted patients affected by BK nephropathy [14]. Based upon the above consideration, BK persistence in humans is allowed by complex viral mechanisms, involving multiple factors and the host defense status. Despite the establishment of durable BK infection in most individuals, significant consequences of BKV infection are uncommon, except for immunocompromised and immunosuppressed patients. Indeed, when the immune system is suppressed (as in RTRs and in transplanted individuals in general), the virus may reactivate and, as a consequence of its replication, trigger a series of effects that begin with tubular cell lysis and the excretion of BKV in urine. Thereafter, BKV replicates in the interstitial cells and crosses the peritubular endothelial barrier, reaching the bloodstream and eventually the allograft, causing different tubular and interstitial damages with consequent serious complications, such as BKVN [15]. BKVN can induce the degeneration of transplanted kidney and graft failure [16], and patient outcome is determined by the severity of injury with consequent inflammatory and fibrosis pictures. Roughly 33–34% of patients with the presence of BKV in urine will develop BK viremia, and could advance to BKVN without intervention.

## 2. Kidney Transplantation

Kidney transplantation is an important epidemiological factor in the general population. It is a lifesaving procedure, and it represents the only alternative to life-long dialysis for patients at the final stage of kidney disfunction (uremia or end-stage renal disease (ESRD)). A total of 80,926 cases of kidney transplantation were reported to the Global Database on Donation and Transplantation (GODT) (http://www.transplant-observatory.org/, accessed on 14 April 2022) in 2020 [17]. A major problem with kidney transplantation is that the transplanted organ may be lost, with the consequent return of patients to an ESRD condition.

One of the main causes (though not the only one) of graft loss is immune rejection, which can have several causes, including BK-virus-related disease. Even though the rate of graft failure has been progressively decreasing over the years, it still represents an important clinical problem. The 2019 Annual Data Report of the Organ Procurement Transplant Network (OPTN)/Scientific Registry of Transplant Recipients (SRTR) reported that 7% of patients receiving a new kidney from a dead donor experienced acute graft rejection by one year, and 5-year graft survival ranged between 85% and 21–35% depending on the value of the Kidney Donor Profile Index (KDPI). In young patients (35–49 years old) transplanted with a kidney from a living donor, the 5-year graft survival is about 90%, whereas it is only 80.2% if the recipient is older than 65 years [18]. Graft failure has large direct medical costs, which have been previously estimated around $78,100 for a single patient, accompanied by a loss of 1.66 quality-adjusted life years (QALYs) [19]. Model extrapolation of these individual data to the total number of kidney transplantations performed in the US in 2017 led to an estimate of costs related to kidney graft failure of about USD 1.38 billion on an annual basis. In this perspective, efforts to improve the efficacy of immunosuppressant pharmacological treatment with the final aim of preserving the graft could contribute to reducing the costs related to this disease.

As mentioned, immune rejection can have various causes, including the reduction of immunosuppressive therapy due to BK-virus-related disease. In addition, BKV induces direct toxic effects on kidney.

## 3. BKV Nephropathy

BKV-associated diseases are usually found in both the donated kidneys and the hematopoietic stem cell of the receiving patients. BKVN is the major BKV-associated disease, and it is defined as persistently viral plasma burden >10,000 copies/mL for 28 days. BKVN manifests in up to 10% of RTR, especially in blood group-incompatible donors and after recipients’ desensitization, with an incidence of rejection between 10% and 80% [20]. Main cause for BKV reactivation consists of therapeutic immunosuppression following transplant [21], but whether the BKV source inducing BKVN is derived from the donor or from recipient reactivation is unclear. From the histological point of view, it is possible to classify BKVN into three stages according to Banff scheme: A, B and C. In stage A, a high cytotoxic effect is followed by a sustained tubulointerstitial inflammation (stage B); the consequences of these events are tubular atrophy and interstitial fibrosis (stage C) [22]. A review of the Banff scheme proposes that the inflammatory state (stage B) and fibrosis (stage C) possess a great importance as prognostic markers. On the other hand, stage A and histologic viral load do not predict an unfavorable outcome [23]. Several risk factors are associated with BKVN development, and certainly the most prevalent is the degree of immunosuppression. Additional identified risk factors for BKVN development are kidneys received from BKV seropositive donors and transplanted to BKV seronegative recipients, the age of both the donor and the recipient [24], obesity, as well as donors and recipients positivity in the sera of both BKV and Cytomegalovirus [25]. Moreover, degree of HLA mismatches, ABO-discordance, and ischemia reperfusion injury are included as risk factors [20]. It was reported recently in a single-center retrospective study the effects of the so-called enhanced induction, (based upon the administration of thymoglobulin, rituximab, and/or eculizumab), together with age, sex, cytomegalovirus mismatch (donor +/recipient−) and transplant failure treatment as risk factors for developing BKVN. Results show that male gender, but not enhanced induction, represents a hazard for developing BKVN [15]. Moreover, in another very recent research work has been assessed the relationship between laboratory data and a higher risk of BKV activation. Results clearly demonstrate that patients with an active BKV infection have higher association with a dead donor, reduced conjugated bilirubin levels, a higher relative percentage of serum albumin, and decreased neutrophil count. These laboratory parameters were used to build a nomogram for predicting BKV activation in RTRs [26]. The main risk factors related to the onset of BKVN are reported in Table 1.

In consideration of the poor efficacy of BKV preventive or curative anti-viral drugs, adopting a heavier immunosuppressive regimen may decrease the risk of BKVN progression. However, these measures increase the risk of transplant failure [27]. On the basis of these considerations, increasing surveillance and early diagnosis would certainly result in more favorable outcomes.

## 4. Screening Tests

The biopsy of kidney allograft is the mainstay for BKVN diagnosis, severity assessment and for concomitant processes evaluation. However, because biopsy is invasive and sampling error can occur, a theoretical diagnosis can be conceived based upon the presence of significant viremia. In order to early recognize BKV infection, screening tests in urine or plasma are recommended, thus allowing intervention and avoiding progression to BKVN or allograft rejection.

Unfortunately, the best periodicity and screening methods are still indefinite. Guidelines suggest intense clinical follow-up investigation in the first year and every 6 months afterwards [28]. Indeed, both the Kidney Diseases Improving Global Outcomes (KDIGO) and the American Society of Transplantation (AST) guidelines suggest the screening of all renal transplants with quantitative real-time PCR testing. Nevertheless, KDIGO recommends screening tests executed on plasma, while AST does not mention which biological matrix (urine or plasma) should be analyzed [29]. BKV DNA detection in plasma using qPCR is the most widely used method to monitor BKV infection, since viruria quite accurately correlates with BKVN. Nevertheless, many studies have emphasized the advantage of BKV screening in urine samples for the prevention of BKVN, as viruria precedes viremia (viremia appears several weeks later due to the tubular viral replication) [27]. A test is considered positive if the viral copies are more than 107 copies/mL in urine and 104 copies/mL in plasma. Similar results must be obtained again within 4 weeks [30]. As mentioned above, the detection of BKV DNA has not been fully standardized. This results in the following several pitfalls: (i) intra- and inter-laboratory assay variability; (ii) significant changes of virus level detection and (iii) assessment technical pitfalls. Variability in biological samples, the techniques for DNA extraction, the primer and probe sequences and different BK DNA used for the construction of standard curves may impact assay results and reduce clinical significance [28,31]. To optimize reproducibility, it is recommended to always perform tests on a certain patient population at the same center to powerfully reduce assay variability. Moreover, only laboratories that work according to good quality control rules and are certified for transplant diagnostics should be considered [32]. The main problem related to BKV DNA detection is that its presence in biological samples is not a marker of an active viral replication. Indeed, viral DNA can also be found when defective virions are shed [27].

For this reason, increasing attention is being paid to the evaluation of certain viral mRNAs as biomarkers for BKV active infection and the prediction of BKVN. Specifically, BKV capsid protein 1 (VP1) mRNA from cells in urinary sediment was assessed as a BKVN marker [33]. Over the past few years, microRNAs (miRNAs) have emerged as encouraging diagnostic and prognostic markers for many diseases, including cancer [34] and viral infections [35]. miRNAs belong to the group of small non-coding RNAs and are formed by about 22 nucleotides; they are implicated in the regulation of gene expression, via either translation inhibition of their target mRNAs or the reduction of their cytoplasmic half-life (mRNA poly-adenylation inhibition) [36]. In detail, miRNAs are secreted by human cells in protective delivery systems (i.e., extracellular vesicles, conjugated to HDL cholesterol or to Ago2 proteins) that increase their circulating half-life in biological fluids, including blood and urine. Moreover, miRNAs are easily and rapidly detectable with relatively cheap conventional qRT-PCR techniques that are widely available in analytical laboratories. Bkv-miR-B13p and bkv-miR-B1-5p are the two miRNAs expressed by BKV, and their roles in the BKV infection cycle are not fully understood. Both bkv-miR-B13p and bkv-miR-B1-5p can cleave large tumor antigen (T-Ag) mRNA, self-regulating viral replication. Moreover, bkv-miR-B1-3p targets ULBP3, thus inducing escape from the immune response. Certainly, bkv-miR-B13p and bkv-miR-B1-5p receiving increasing interest for the detection of BKV and the diagnosis of active infection in RTRs [16,27]. It has also been found that BKV miRNAs are expressed in different biological fluids such as blood, urine and cerebrospinal fluid [37]. Moreover, urine levels of BKV miRNAs correlate with BKV DNA load in RTRs [38]; for this reason, bkv-miR-B13p and bkv-miR-B1-5p represent non-invasive diagnostic biomarkers for BKV. Nevertheless, few results on the use of BKV miRNAs as markers of active BKV infection are available, and they are not easy to compare. In fact, the biological samples (cell pellets, native urine or exosomes) and standardization methods differ among laboratories. Therefore, taking into account the potential diagnostic advantages related to urine miRNAs as BKV infection biomarkers, it appears worthwhile to conduct studies in this field.

## 5. Treatments

There is currently no specific anti-viral therapy to treat BKV-associated diseases. Indeed, the usual clinical approach consists of a gradual reduction of immunosuppression, guided by consecutive measurements of BKV presence in plasma by qRT-PCR. However, the main consequence of a long-term reduction of immunosuppression is an increased number of patients suffering from chronic rejection. Despite the lack of specific anti-BKV medications, some anti-viral drugs that are efficient in CMV disease have been employed in BKV-related pathologies and associated with immunosuppression downmodulation. However, most of the studies showing the application of the abovementioned anti-viral drugs were uncontrolled retrospective observational studies. For this reason, the therapeutic efficacy of anti-CMV viral agents in BKV-associated disease is not easy to confirm [8,39]. Recent guidelines suggest stepwise immunosuppression reduction for kidney transplanted patients with BK viremia of more than 1000 copies/mL lasting for 3 weeks, or a one-shot detection of more than 10,000 copies/mL in sera, showing a probable BKVN. The reduction of the immunosuppressive schedules is the most important intervention for BKVN proven in kidney tissue [40]. In cases of refractory BK nephropathy and hemorrhagic cystitis, cidofovir has been used for treatment (IV and intra-vesicular), although efficacy has not been clearly demonstrated [41]. Another study suggests that cidofovir could be effective for BKV-related hemorrhagic cystitis [42]. Nevertheless, its employment needs to be supported by randomized controlled trials. Adoptive immune transfer of BKV-specific T cells has been anecdotally explored to treat hemorrhagic cystitis [43].

Fluoroquinolones also show potential as anti-viral agents against BKV-associated disease. Indeed, it was recently demonstrated that this class of antibiotics restrain BKV replication in vitro [44]. However, data on this class of antibiotics are still inconsistent. A phase III clinical trial involving 154 Canadian kidney transplanted patients demonstrated that levofloxacin, and likely other fluoroquinolones, are ineffective in preventing or treating this infection [45]. Recent guidelines state that the latter antibiotics are not recommended for prophylaxis or therapy [40].

In conclusion, despite the virological basis, the published randomized clinical trials are not adequate to replace the immunosuppressant therapy (tacrolimus with cyclosporine A and mycophenolate with leflunomide or mTOR inhibitors). Moreover, they do not legitimize the additive use of cidofovir, intravenous immunoglobulins or leflunomide [40].

Re-transplantation after allograft rejection due to BKVN may be successful if BKV DNAemia is completely cleared, independent of failed allograft nephrectomy [40].

Other adjunctive therapies with unproven efficacy include intravenous immune globulin and leflunomide. In fact, the efficacy of these agents has not been fully established, and the use of these therapies has not been clearly shown to be superior to reduction in immunosuppression alone.

## 6. Animal Models to Study BKVN

The development of animal models to study BKV infection and associated nephropathy is made difficult by the narrow host range and cell specificity of BKV and other PVs. Despite these considerations, the main difficulties in obtaining a mouse animal model for studying BKV infection are related to mouse-specific genetic background and the promoter choice driving the expression of the transgene and strongly influencing disease phenotype [46]. One example is represented by transgenic mice containing the early region of BKV used to study the role of T-Ag in the pathogenesis of BKVN. These transgenic mice developed primary hepatocellular carcinomas and renal tumors, but did not develop key features of BKVN [47]. To overcome these problems, researchers assessed mouse BKV infection in mice bearing allogeneic kidneys to mimic BKVN. In this model, infection with the mouse BKV resulted in a high viral replication in the allogeneic kidney graft, severe graft injury and accelerated kidney graft failure [48]. However, results from this mouse model were poorly associated with human RTRs. Indeed, the recipient mice were not immunosuppressed because immuno-competent mice did not acutely reject allogeneic kidneys. To address this issue, kidney transplantations in splenectomized and nephrectomized alymphoplasia mice was performed [49]. Although high viral loads were observed in transplanted mice, they were not associated with increased allograft injury or loss of renal grafts, suggesting that BKVN in mice is dependent on an intact adaptative immune response [46]. Further studies are necessary to build an appropriate animal model to study BKVN in humans. Other strategies could be represented by xenograft or humanized mouse models.

## 7. Conclusions

BKV infection is recorded in up to 90% of the general population. It can be found as permanent and latent states of infection in renal cells and uroepithelium. BKV reactivation is commonly observed in immunocompromised individuals, causing relevant morbidity, especially BKVN, in RTRs. No specific therapeutic intervention is available, and its treatment is generally based upon immunosuppression decrease. To date, only increasing surveillance and early diagnosis have resulted in more favorable outcomes. The available screening tests are based on BKV DNA detection, and give no information about the active viral replication. On the other hand, BKV miRNAs quantification could represent a new effective strategy to accomplish early diagnosis, as well as better RTR surveillance and management.

## Figures and Tables

**Table 1:** Principal Risk Factors for the onset of BKVN.

Donors Factors	Host Factors	Transplant Factors
BKV Seropositive [24]	Advanced Age [24]	Ischemia Reperfusion [20]
Age [24] Deceased [26]	Male Gender [15] Obesity [25] Diabetes [8] Low Direct Bilirubin High Serum Albumin [26] Low Neutrophil Count [26]	Degree of HLA Mismatch [20] ABO Incompatibility [20] Bilirubin [26]

## Data Availability

Not applicable.

## References

[1] Sharma R., Zachariah M. (2020). BK Virus Nephropathy: Prevalence, Impact and Management Strategies. Int. J. Nephrol. Renov. Dis..

[2] Takasaka T., Goya N., Tokumoto T., Tanabe K., Toma H., Ogawa Y., Hokama S., Momose A., Funyu T., Fujioka T. (2004). Subtypes of BK virus prevalent in Japan and variation in their transcriptional control region. J. Gen. Virol..

[3] Hirsch H.H., Steiger J. (2003). Polyomavirus BK. Lancet Infect. Dis..

[4] Chatterjee M., Weyandt T.B., Frisque R.J. (2000). Identification of archetype and rearranged forms of BK virus in leukocytes from healthy individuals. J. Med. Virol..

[5] Knowles W.A. (2006). Discovery and epidemiology of the human polyomaviruses BK virus (BKV) and JC virus (JCV). Adv. Exp. Med. Biol..

[6] Chesters P.M., Heritage J., McCance D.J. (1983). Persistence of DNA sequences of BK virus and JC virus in normal human tissues and in diseased tissues. J. Infect. Dis..

[7] Goudsmit J., Wertheim-van Dillen P., van Strien A., van der Noordaa J. (1982). The role of BK virus in acute respiratory tract disease and the presence of BKV DNA in tonsils. J. Med. Virol..

[8] Jamboti J.S. (2016). BK virus nephropathy in renal transplant recipients. Nephrology.

[9] Pastrana D.V., Ray U., Magaldi T.G., Schowalter R.M., Çuburu N., Buck C.B. (2013). BK Polyomavirus Genotypes Represent Distinct serotypes with Distinct Entry Tropism. J. Virol..

[10] Gerits N., Moens U. (2012). Agnoprotein of mammalian polyomaviruses. Virology.

[11] Rinaldo C.H., Traavik T., Hey A. (1998). The Agnogene of the Human Polyomavirus BK Is Expressed. J. Virol..

[12] Unterstab G., Gosert R., Leuenberger D., Lorentz P., Rinaldo C.H., Hirsch H.H. (2010). The polyomavirus BK agnoprotein co-localizes with lipid droplets. Virology.

[13] Panou M.M., Prescott E.L., Hurdiss D.L., Swinscoe G., Hollinshead M., Caller L.G., Morgan L., Carlisle L., Müller M., Antoni M. (2018). Agnoprotein Is an Essential Egress Factor during BK Polyomavirus Infection. Int. J. Mol. Sci..

[14] Manzetti J., Weissbach F.H., Graf F.E., Unterstab G., Wernli M., Hopfer H., Drachenberg C.B., Rinaldo C.H., Hirsch H.H. (2020). BK Polyomavirus Evades Innate Immune Sensing by Disrupting the Mitochondrial Network and Promotes Mitophagy. iScience.

[15] Lorant C., Westman G., Bergqvist A., von Zur-Mühlen B., Eriksson B.M. (2022). Risk Factors for Developing BK Virus-Associated Nephropathy: A single-center retrospective cohort study of kidney transplant recipients. Ann. Transplant..

[16] Kim M.H., Lee Y.H., Seo J.W., Moon H., Kim J.S., Kim Y.G., Jeong K.H., Moon J.Y., Lee T.W., Ihm C.G. (2017). Urinary exosomal viral microRNA as a marker of BK virus nephropathy in kidney transplant recipients. PLoS ONE.

[17] Global Observatory Donation and Transplantation. http://www.transplant-observatory.org/.

[18] Hart A., Singh D., Brown S.J., Wang J.H., Kasiske L. (2021). Incidence, risk factors, treatment, and consequences of antibody-mediated kidney transplant rejection: A systematic review. Am. J. Transplant..

[19] Sussell J., Silverstein A.R., Goutam P., Incerti D., Kee R., Chen C.X., Batty D.S., Jansen J.P., Kasiske B.L. (2020). The economic burden of kidney graft failure in the United States. Am. J. Transplant..

[20] Sharif A., Alachkar N., Bagnasco S., Geetha D., Gupta G., Womer K., Arend L., Racusen L., Montgomery R., Kraus E. (2012). Incidence and Outcomes of BK Virus Allograft Nephropathy among ABO- and HLA-Incompatible Kidney Transplant Recipients. Clin. J. Am. Soc. Nephrol..

[21] Leung A.Y., Chan M., Tang S.C., Liang R., Kwong Y. (2002). Real-time quantitative analysis of polyoma BK viremia and viruria in renal allograft recipients. J. Virol. Methods.

[22] Sis B., Mengel M., Haas M., Colvin R.B., Halloran P.F., Racusen L.C., Solez K., Baldwin W.M., Bracamonte E.R., Broecker V. (2010). Banff ’09 meeting report: Antibody mediated graft deterioration and implementation of banff working groups. Am. J. Transplant..

[23] Masutani K., Shapiro R., Basu A., Tan H., Wijkstrom M., Randhawa P. (2012). The Banff 2009 Working Proposal for Polyomavirus Nephropathy: A critical evaluation of its utility as a determinant of clinical outcome. Am. J. Transplant..

[24] Hirsch H.H., Randhawa P. (2013). BK Polyomavirus in Solid Organ Transplantation. Am. J. Transplant..

[25] Borni-Duval C., Caillard S., Olagne J., Perrin P., Braun-Parvez L., Heibel F., Moulin B. (2013). Risk factors for BK virus infection in the era of therapeutic drug monitoring. Transplantation.

[26] Wang J., Li J., Chen Z., Xu M., Yang C., Rong R., Zhu T. (2022). A Nomogram for Predicting BK Virus Activation in Kidney Transplantation Recipients Using Clinical Risk Factors. Front. Med..

[27] Demey B., Descamps V., Presne C., Helle F., Francois C., Duverlie G., Castelain S., Brochot E. (2021). BK Polyomavirus Micro-RNAs: Time course and clinical relevance in kidney transplant recipients. Viruses.

[28] Sawinski D., Trofe-Clark J. (2018). BK Virus Nephropathy. Clin. J. Am. Soc. Nephrol..

[29] Brochot E., Descamps V., Handala L., Faucher J., Choukroun G., Helle F., Castelain S., François C., Duverlie G., Touzé A. (2019). BK polyomavirus in the urine for follow-up of kidney transplant recipients. Clin. Microbiol. Infect..

[30] Zakaria Z.E., Elokely A.M., Ghorab A.A., Bakr A.I., Halim M.A., Gheith O.A., Nagib A.M., Makkeyah Y., Balaha M.A., Magdy M.M. (2019). Screening for BK Viremia/Viruria and the Impact of Management of BK Virus Nephropathy in Renal Transplant Recipients. Exp. Clin. Transplant..

[31] Trofe-Clark J., Sparkes T., Gentile C., Van Deerlin V., Sawinski D., Bloom R.D. (2013). BK Virus Genotype Variance and Discordant BK Viremia PCR Assay Results. Am. J. Transplant..

[32] Hirsch H.H., Babel N., Comoli P., Friman V., Ginevri F., Jardine A., Lautenschlager I., Legendre C., Midtvedt K., Munoz P. (2014). ESCMID Study Group of Infection in Compromised Hosts: European perspective on human polyomavirus infection, replication and disease in solid organ transplantation. Clin. Microbiol. Infect..

[33] Sawinski D., Goral S. (2015). BK virus infection: An update on diagnosis and treatment. Nephrol. Dial. Transplant..

[34] Grimaldi A., Zarone M., Irace C., Zappavigna S., Lombardi A., Kawasaki H., Caraglia M., Misso G. (2018). Non-coding RNAs as a new dawn in tumor diagnosis. Semin. Cell Dev. Biol..

[35] Gilad S., Meiri E., Yogev Y., Benjamin S., Lebanony D., Yerushalmi N., Benjamin H., Kushnir M., Cholakh H., Melamed N. (2008). Serum MicroRNAs Are Promising Novel Biomarkers. PLoS ONE.

[36] Fourdinier O., Schepers E., Meuth V.M.-L., Glorieux G., Liabeuf S., Verbeke F., Vanholder R., Brigant B., Pletinck A., Diouf M. (2019). European Uremic Toxin Work Group-EUTox. Serum levels of miR-126 and miR-223 and outcomes in chronic kidney disease patients. Sci. Rep..

[37] Pietilä T., Nummi M., Auvinen P., Mannonen L., Auvinen E. (2015). Expression of BKV and JCV encoded microRNA in human cerebrospinal fluid, plasma and urine. J. Clin. Virol..

[38] Huang Y., Zeng G., Randhawa P.S. (2019). Detection of BKV encoded mature MicroRNAs in kidney transplant patients: Clinical and biologic insights. J. Clin. Virol..

[39] Ambalathingal G.R., Francis R.S., Smyth M.J., Smith C., Khanna R. (2017). BK Polyomavirus: Clinical aspects, immune regulation, and emerging therapies. Clin. Microbiol. Rev..

[40] Hirsch H.H., Randhawa P.S. (2019). AST Infectious Diseases Community of Practice. BK polyomavirus in solid organ transplantation-Guidelines from the American Society of Transplantation Infectious Diseases Community of Practice. Clin. Transplant..

[41] Kuypers D.R., Vandooren A.K., Lerut E., Evenepoel P., Claes K., Snoeck R., Naesens L., Vanrenterghem Y. (2005). Adjuvant Low-Dose Cidofovir Therapy for BK Polyomavirus Interstitial Nephritis in Renal Transplant Recipients. Am. J. Transplant..

[42] Cesaro S., Hirsch H.H., Faraci M., Owoc-Lempach J., Beltrame A., Tendas A., Baltadakis I., Dalle J.H., Koc Y., Toporski J. (2009). European Group for Blood and Marrow Transplantation. Cidofovir for BK virus-associated hemorrhagic cystitis: A retrospective study. Clin. Infect. Dis..

[43] Pello O.M., Innes A.J., Bradshaw A., Finn S.A., Uddin S., Bray E., Olavarria E., Apperley J.F., Pavlů J. (2017). BKV-specific T cells in the treatment of severe refractory haemorrhagic cystitis after HLA-haploidentical haematopoietic cell transplantation. Eur. J. Haematol..

[44] Sharma B.N., Li R., Bernhoff E., Gutteberg T.J., Rinaldo C.H. (2011). Fluoroquinolones inhibit human polyomavirus BK (BKV) replication in primary human kidney cells. Antivir. Res..

[45] Knoll G.A., Humar A., Fergusson D., Johnston O., House A.A., Kim S.J., Ramsay T., Chassé M., Pang X., Zaltzman J. (2014). Levofloxacin for BK Virus Prophylaxis Following Kidney Transplantation: A Randomized Clinical Trial. JAMA.

[46] Barth H., Solis M., Kack-Kack W., Soulier E., Velay A., Fafi-Kremer S. (2016). In Vitro and In Vivo Models for the Study of Human Polyomavirus Infection. Viruses.

[47] Moriyama T., Marquez J.P., Wakatsuki T., Sorokin A. (2007). Caveolar Endocytosis Is Critical for BK Virus Infection of Human Renal Proximal Tubular Epithelial Cells. J. Virol..

[48] Lee E.D.H., Kemball C.C., Wang J., Dong Y., Stapler D.C., Hamby K.M., Gangappa S., Newell K.A., Pearson T.C., Lukacher A.E. (2006). A Mouse Model for Polyomavirus-Associated Nephropathy of Kidney Transplants. Am. J. Transplant..

[49] Albrecht J.A., Dong Y., Wang J., Breeden C., Farris A.B., Lukacher A.E., Newell K.A. (2012). Adaptive immunity rather than viral cytopathology mediates polyomavirus-associated nephropathy in mice. Am. J. Transplant..

